# Supplementary material for: The respiratory microbiome in pulmonary tuberculosis: a meta-analysis reveals niche-specific microbial and functional signatures
Source: mSystems. 2026 Mar 25;11(4):e01563-25. doi: 10.1128/msystems.01563-25 (PMC13098208; doi:10.1128/msystems.01563-25)
Supplement: Legends — Supplemental figure legends. [file msystems.01563-25-s0002.docx]

**Figure S1.** The improvement effect of batch effect correction (ConQuR method) on the data structure was demonstrated and evaluated using the Bray-Curtis distance

**Figure S2. Altered diversity of the respiratory microbiome between PTB and HC group.** (A) Alpha diversity comparisons between PTB patients and HC within each respiratory samples, including the URT, sputum, and BALF, assessed using Shannon, Simpson, Chao1, and ACE indices. Boxplots show medians and interquartile ranges, two-sided Wilcoxon rank-sum tests were used for statistical comparisons. (B) PCoA based on Bray–Curtis dissimilarity showing differences in microbial community structure between PTB and HC across respiratory samples. Group-level differences were assessed using PERMANOVA.

**Figure S3. The LEfSe difference genera analysis of each sample in the PTB and HC group.** LEfSe results identifying taxa with statistically significant differences in abundance among (A) URTs, (B) sputum, and (C) BALF samples. The histogram shows the LDA scores (log10) for taxa enriched in each specific sample type, with only scores exceeding a significant threshold displayed. The size of the bubbles indicates the level of abundance.

**Figure S4. Heatmap showing the comparisons between groups for the top 50 KEGG pathways.** The heatmap presents a statistical comparison of the relative abundance of the top 50 most enriched microbial KEGG pathways across URT, Sputum and BALF samples. Pairwise comparisons between all three groups were performed using the rank-sum test. The color of each data point represents the fold change in the abundance of the metabolic pathway between the compared groups. Statistical significance is denoted by asterisks: *p < 0.05, **p < 0.01, ***p < 0.001.
