# Supplementary material for: The respiratory microbiome in pulmonary tuberculosis: a meta-analysis reveals niche-specific microbial and functional signatures
Source: mSystems. 2026 Mar 25;11(4):e01563-25. doi: 10.1128/msystems.01563-25 (PMC13098208; doi:10.1128/msystems.01563-25)
Supplement: Table S1 — Detailed data information of healthy control group. [file msystems.01563-25-s0003.docx]

| Year | **Dataset** | **Platform** | **Data type** | **Site** | **Sample type (n)** | **Sample origin** |
| --- | --- | --- | --- | --- | --- | --- |
| 2014 | PRJNA242354 | 454 | V1-V2 | URT | nasal (6), oropharynx (6) | Columbia |
| 2018 | PRJNA432583 | Miseq | V3-V4 | URT | nasopharyngeal and oropharyngeal swabs (5) | Myanmar |
| 2022 | SRP368119 | Miseq | V3-V4 | Lung | BALF (13) | China |
| 2022 | PRJNA837186 | HiSeq | V1-V3 | Airway | Sputum (30) | China |
| 2024 | <https://doi.org/10.5281/>  zenodo.11560386 | Miseq | V3-V4 | Airway | Sputum (16) | India |

Table S1. The detailed information of the original sample data for healthy controls.
